# Clinical findings, outcomes following management and complications of acute retinal necrosis: the experience of a tertiary eye centre in Saudi Arabia

**DOI:** 10.1186/s12348-025-00511-8

**Published:** 2025-07-01

**Authors:** Enas Magharbil, Faisal Al-Qahtani, Maram Al-Enazi, Abdulrahman H. Badawi, Nora Alyousif, Moustafa S. Magliyah, Hassan Al-Dhibi

**Affiliations:** 1 Vitreoretinal and Uveitis Division, King Khaled Eye Specialist Hospital, PO Box 7141, Riyadh, 11462 Saudi Arabia; 2 Uveitis department, Jeddah Eye Hospital, Jeddah, Saudi Arabia; 3 Medical Education Department, Alfaisal University, Riyadh, Saudi Arabia

**Keywords:** ARN, Acute retinal necrosis, Viral retinitis, Panuveitis, Retinal detachment, Pars plana vitrectomy, Scleral bucking

## Abstract

**Purpose:**

To study the clinical picture, outcomes and the complications of acute retinal necrosis (ARN) in a tertiary eye hospital.

**Methods:**

This is retrospective chart review of all patients who were diagnosed with ARN and were treated and followed up at King Khaled Eye Specialist Hospital (KKESH). Details of clinical examinations, Polymerase Chain Reaction (PCR) results, systemic and topical treatments, complications and managements of complications were obtained.

**Results:**

Twenty eight eyes of 26 patients were included. The results of PCR were positive in 22 eyes. Systemic antiviral therapy with intravenous Acyclovir 10 mg/Kg three times daily was given to all cases. Eight patients were treated with intravitreal ganciclovir injections. Fifteen eyes (56.3%) had rhegmatogenous retinal detachment (RRD) and 11 of them had surgical interventions. The risk of developing RRD was significantly high in severe vitritis (*P* = 0.007, OR = 3.825), diffuse or multifocal retinitis (*P* = 0.010, OR = 1.04) and the larger extent of retinitis (*P* = 0.016). The final visual outcome was worse among eyes which developed RRD (LogMAR 1.6 ± 0.94, Snellen = 20/800) than eyes which did not develop RRD (LogMAR 0.81 ± 0.84, Snellen = 20/125) and the difference was statistically significant (*P* = 0.031).

**Conclusion:**

The visual outcomes of ARN are significantly worse in eyes which develop RRD. More severe and larger extent of posterior segment involvement confer higher risks of RRD development in ARN.

**Supplementary Information:**

The online version contains supplementary material available at 10.1186/s12348-025-00511-8.

## Key summary bullet points

• Acute retinal necrosis (ARN) is a serious retinal condition which can lead to permanent and severe visual loss.

• ARN can result in severe ophthalmic complications including rhegmatogenous retinal detachment (RRD) in 30–75% of cases.

• The risk factors for RRD development are not well delineated in the literature and are discussed in detail in this paper.

• The long-term outcomes of RRD in ARN and details of surgical repair are also provided in this study.

## Introduction

Acute retinal necrosis (ARN) is a serious and progressive ophthalmic emergency which manifests as posterior uveitis. It was first described in 1971 by Urayama, a Japanese ophthalmologist who reported.

panuveitis, retinitis and retinal arteritis that ultimately led to retinal detachment and had poor outcomes [1]. The most common etiologies of ARN include varicella-zoster virus (VZV), herpes simplex virus 1 and 2 (HSV-1 and 2), and cytomegalovirus (CMV) [2]. ARN typically affects immunocompetent patients, unlike Progressive Outer Retinal Necrosis (PORN), the herpetic retinopathy of the immunocompromised [3]. The revised classification criteria of ARN approved by the Executive Committee of the Standardization of Uveitis Nomenclature (SUN) Working Group in 2021 are [4]:

1. Necrotizing retinitis involving the peripheral retina AND (either #2 OR #3).
2. Evidence of infection with HSV or VZV through Polymerase Chain Rearction (PCR) testing.
3. Characteristic clinical picture of Circumferential or confluent retinitis, vascular sheathing and/or occlusion and + 1 or more vitritis.

A prompt initiation of systemic antiviral therapy is considered the mainstay of management of ARN and to reduce the risk of contralateral eye involvement [5, 6]. In addition, systemic and topical steroids therapy is initiated to halt the progression of retinitis and reduce the 30–75% risk of rhegmatogenous retinal detachment (RRD) [7, 8]. The clinical features and outcomes of ARN in Saudi Arabia and the gulf region are not documented in the literature.

In this study we describe the clinical picture, management outcomes, and complications of ARN in a tertiary eye center in Saudi Arabia. In addition, we try to identify the risk factors and management outcomes in eyes with RRD.

## Methods

### Patient selection and study population

This study is a retrospective chart review design of patients with ARN in King Khaled Eye Specialist Hospital (KKESH) who were diagnosed and treated between 2014 and 2024. Cases were identified from electronic hospital records database through the search terms “Acute retinal necrosis” and “Retinitis”. The criteria defined by the revised classification criteria of ARN approved by the Executive Committee of the SUN Working Group were used for inclusion [4]. This study adhered to the tenets of the declaration of Helsinki at 1964 and its amendments. The study was approved by the Institutional review board at KKESH. Informed consents were obtained from all participants included in this study.

### Data collection

Electronic medical records were retrospectively reviewed to obtain the following: age at presentation, sex, history of a herpetic infection, chief compliant, laterality, duration of symptoms prior to presentation, and previous medical history including the immune status of the patient. The following ophthalmologic finding were collected: initial and final best-corrected visual acuity (BCVA) (using Snellen chart and was converted to LogMAR during statistical analysis); presence or absence of keratic precipitate, grade of anterior chamber reaction, iris atrophy, posterior synechiae, lens status; After dilated fundus exam, grade of vitritis, initial clock hours of retinal quadrants involved, vascular sheathing and optic disc swelling or hyperemia were defined; and occurrence of complications like Band keratopathy, glaucoma and RRD. The grade of vitritis was considered mild if it was 1 + or less, and severe if it was 3 + or more. Diffuse retinitis was defined as 3 or more clock hours of confluent retinitis lesions on examination. Aqueous PCR results were collected from patients’ electronic records whenever available. Type of systemic antiviral medication and intravitreal injections, dose, frequency and duration also were collected.

Exclusion criteria were other causes of uveitis as tuberculosis (TB), syphilis, toxoplasmosis and Behcet’s disease. In addition, cases which had less than 6 months of follow up were excluded. Cases of TB were excluded based on positive purified protein derivative (PPD) and TB Quantiferon tests with or without signs of pulmonary TB on chest computed tomography. Cases of syphilis were excluded based on positive venereal disease research laboratory (VDRL) and fluorescent treponemal antibody absorption (FTA-ABS). Behcet’s disease cases were excluded based on the positive history and clinical findings of recurrent oral ulcers or genital ulcers or skin nodules with or without positive human leukocyte antigen B51 (HLA-B51) testing.

### Main outcome measures

The main outcome measures included delineating the clinical features, of ARN, the visual outcomes, the complications of ARN and management of these complications.

Secondary outcome measures included looking for factors and features which were associated with the development of RRD and worse visual outcomes.

### Data analysis

Data were analyzed using IBM SPSS Statistics version 26.0 (IBM, Armonk, NY, USA). Frequencies and percentages have been computed to describe categorical data. Means with standard deviations or median with IQR computed to describe continuous data. Chi-squared test was used to compare categorical data and analysis of variance (ANOVA) was used for comparison of numerical data. A *P*-value of < 0.05 will be considered significant. The multivariate regression analysis was used to investigate the predictive value of the possible variables on the occurrence of RRD, and to investigate the predictive value of possible variables on the visual outcomes.

## Results

### Patient demographics

Twenty eight eyes of 26 patients were included. Two eyes of 2 patients which had less than 6 months of follow ups were excluded. Of the 26 patients included, 16 (61.5%) were males and 10 (38.5%) were females. The average age on presentation was 44.64 ± 14.4 years. Twenty four patients had (92.3%) unilateral ARN, and 2 patients had bilateral ARN. The average duration of follow up was 7.1 ± 6.1 years. Right and left eye were equally involved (50.0%) each. Only 2 patients were immunocompromised; one had renal transplantation while the other had human immunodeficiency virus (HIV) infection. 13 patients (50%) had diabetes mellitus, and 7 patients (26.9%) had hypertension. 23 patients (88.5%) complained of decreased vision, 21 patients (80.8%) complained of floaters, 13 patients (50%) complained of eye pain and redness and 6 patients (23.1%) complained of photophobia. Four eyes (14.3%) had previous phacoemulsification with intraocular lens implantation. The average duration of complaints was 14.1 ± 8.9 days. The average visual acuity on presentation was 1.2 ± 0.9 (Snellen = 20/300). The average intraocular pressure (IOP) was 16.7 ± 5.3 mmHg. The average axial length was 23.43 ± 0.69 mm. The details of clinical examination are found in Table 1. The average extent of retinitis was 9.9 ± 3.9 clock hours. All patients underwent essential basic uveitis work-up. Twenty-two eyes (78.6%) underwent anterior chamber tap for polymerase chain reaction (PCR) to detect viral etiologies which revealed that VZV was detected in 15 (68.2%), HSV1 in 7 (31.8%) eyes. The average age of patients who tested positive for HSV1 was 43.8 years, while patients who tested positive for VZV were 41.8 years of age. The difference was statistically not significant (*P* = 0.623).

**Table 1:** Clinical examination findings in 28 eyes presented with Acute retinal necrosis

Clinical examination findings	Number of eyes among 28 eyes (%)
Anterior chamber reaction	
1+	10 (35.7)
2+	9 (32.1)
3+	8 (28.6)
4+	1 (3.6)
Keratic percipitates (KPs)	
Yes	13 (46.4)
No	15 (53.6)
Vitritis	
1+	3 (10.7)
2+	9 (32.1)
3+	11 (39.3)
4+	5 (17.9)
Phakic status	
Phakic	23 (82.1)
Pseudophakic	4 (14.3)
Aphakic	1 (3.6)
Lens status (among 23 phakic eyes)	
Clear	18 (78.3)
Cataract	5 (21.7)
Retinitis	
Focal	4 (14.3)
Multifocal	7 (25)
Diffuse	17 (60.7)
Vasculitis	
Peripheral only	22 (78.6)
Peripheral and posterior	6 (21.4)
Polymerase chain reaction (PCR) test	
Yes	22 (78.6)
No	6 (21.4)
PCR test result (among 22 eyes which had positive tests)	
HSV1	7 (31.8)
VZV	15 (68.2)

### Treatment

Systemic antiviral therapy with intravenous Acyclovir 10 mg/Kg TID was given to all cases (100%) ranging between 3 and 14 days with a median of 7 days. One patient developed allergic reactions at the site of intravenous acyclovir and 2 patients noticed to have increase in the level urea and creatinine which resulted in discontinuation of the intravenous acyclovir and the initiation of oral valacyclovir. In addition, 8 patients were treated with intravitreal ganciclovir antiviral therapy. The decision of intravitreal antiviral treatment was physician dependent and was tailored according to the severity of the clinical picture on presentation. The details of intravitreal antiviral therapy are summarized in Table 2. Systemic steroid (1 mg/kg/day tapering) and topical steroids were added to all patients after 3–5 days from the initiation of intravenous acyclovir to control inflammation and were slowly tapered. All patients were prescribed oral valacyclovir 1000 mg three times daily with slow tapering over 6 to 24 months as prophylactic regimens to reduce the risk of recurrence or contralateral involvement. Renal function testing was performed every 6–8 weeks throughout the duration of prophylactic management.

**Table 2:** Intravitreal antiviral management of 28 eyes with ARN

Intravitreal management	Number of eyes (%)
Intravitreal antiviral injection (*n*=28)	
Yes, Ganciclovir	8 (28.6)
No	20 (71.4)
Frequency of intravitreal injection (*n*=8)	
Once	1 (12.5)
Once/week	3 (37.5)
Twice/week	4 (50)
Duration of intravitreal injection (*n*=8)	
One week	2 (25)
2–3 weeks	4(50)
4–6 weeks	2(25)

### Complications and RRD

**Fig. 1:**
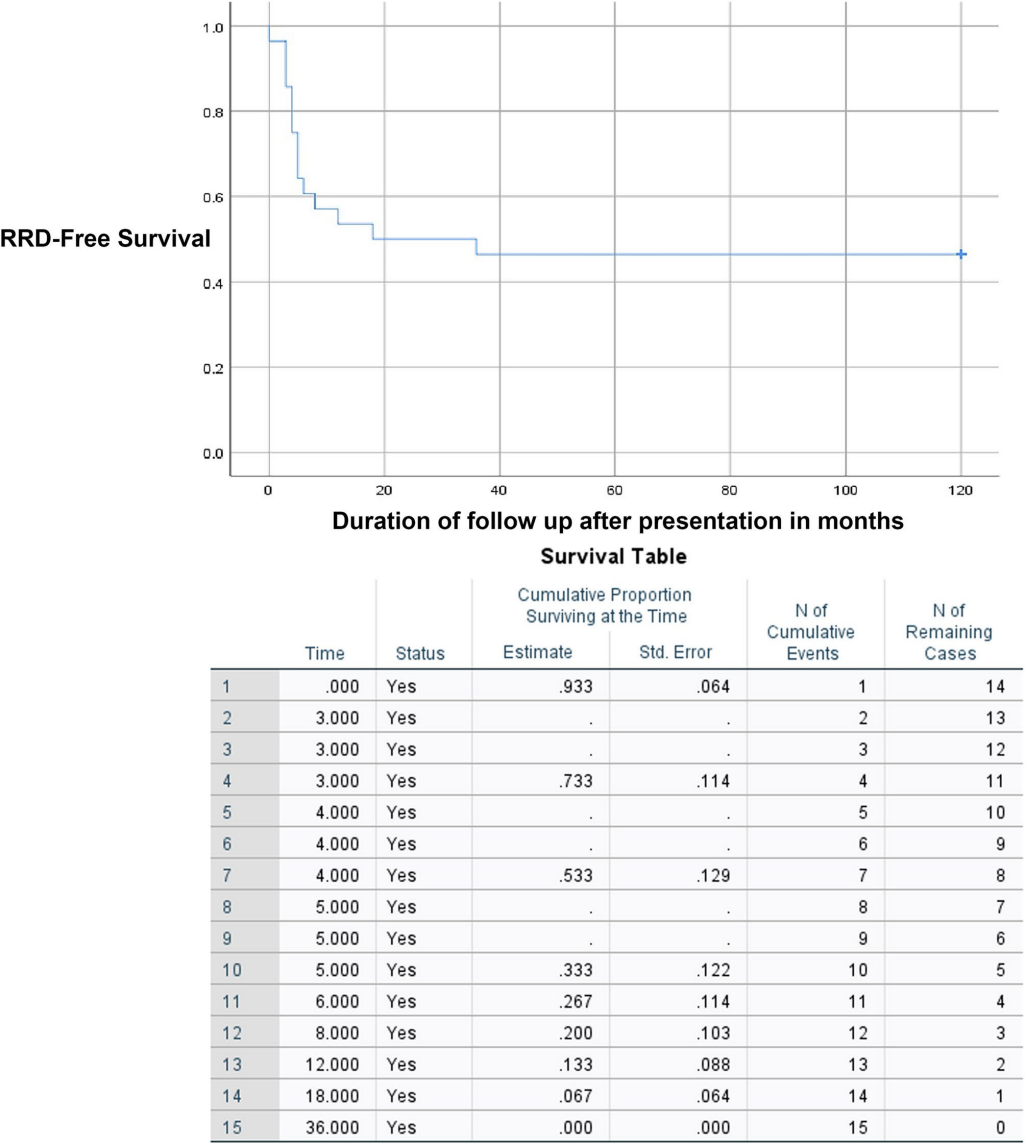
Kaplan Meier survival curve of the probability of acute retinal necrosis (ARN) eyes not developing RRD over time

**Fig. 2:**
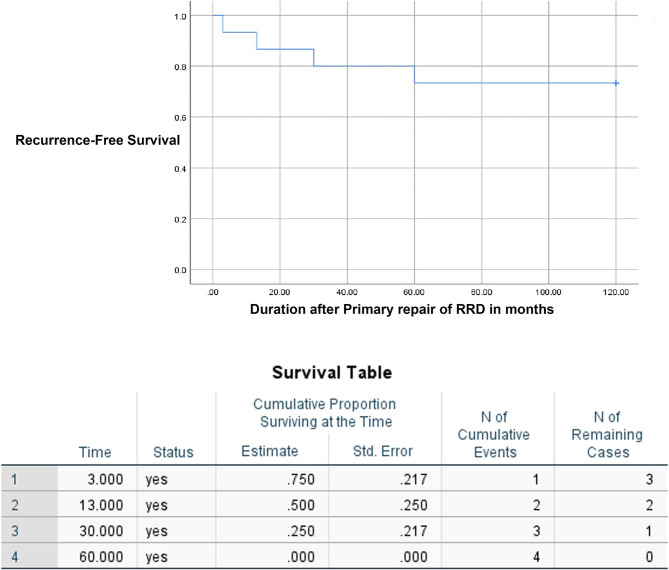
Kaplan Meier survival curve of eyes being free of recurrent RRD after primary repair

Sixteen eyes (57.1%) had ocular complications which could require interventions during the course of follow ups and prophylactic managements after the completion of intravenous acyclovir treatment. Prophylactic laser treatment was performed on 12 (42.9%) eyes. 15 eyes (56.3%) had RRD, including 7 out of 12 eyes that underwent prophylactic laser (58.3%). The average duration between RRD and hospital presentation was 7.7 ± 8.9 days. Figure 1 shows the Kaplan Meier survival curve of the probability of ARN eyes not developing RRD over time. Among 15 eyes which had RRD, 11 eyes had surgical repairs, while 4 eyes were deemed inoperable. The details of surgical management of RRD are shown in Table 3. Eight out of 9 eyes with silicone oil had silicone oil removal after 11 ± 3.7 months. The risk of developing RRD was significantly higher in eyes which had higher grades of vitritis (*P* = 0.007, OR = 3.825), diffuse retinitis (*P* = 0.010, OR = 1.04) and the extent of retinitis (*P* = 0.016). The survival analysis in relation to the grades of vitritis and severity of retinitis are shown in supplemental Figs. 1 and 2. There was a marginally significant relationship between the administration of intravitreal antiviral injection and the risk of RRD (*P* = 0.055). Four eyes (36.4%) had recurrent RRD after 26.5 ± 25 months (3–60 months), while 7 eyes remained attached (63.6%). Figure 2 shows the Kaplan Meier survival curve of eyes being free of recurrent RRD after primary repair. All 4 eyes were reattached after additional repairs. Table 4 shows the analysis of possible risk factors for RRD in ARN. Two eyes (7.1%) had central retinal artery occlusions and 2 further eyes (7.1%) developed glaucoma. The final visual outcome was worse among eyes which developed RRD (LogMAR 1.6 ± 0.94, Snellen = 20/800) than eyes which did not develop RRD (LogMAR 0.81 ± 0.84, Snellen = 20/125) and the difference was statistically significant (*P* = 0.031). Factors which were significantly predictive of visual outcomes include the male gender (*P* = 0.035), initial visual acuity (*P* = 0.009) and RRD (*P* = 0.031). Table 5 shows analysis of possible risk factors for worse visual outcomes.

**Table 3:** Management of RRD in eyes with ARN

Management of eyes with RRD	Eyes (*n*=15).
Surgical intervention	
Yes	11 (73.3%)
No	4 (26.7%)
Type of Surgery	
PPV	6 (54.5%)
SB + PPV	3 (27.3%)
Phaco + PCIOL + SB + PPV	1 (9.1%)
PPL + PPV + SB	1 (9.1%)
Tamponade	
Silicone oil	9 (81.8%)
Air	2 (18.2%)
Silicone oil removal	
Yes	8 (88.9%)
No	1 (11.1%)

**Table 4:** Analysis of possible risk factors for RRD in ARN

Feature	Eyes without RRD (*n*=13)	Eyes with RRD (*n*=15)	Significance	Odds ratio (OR)
Duration of eye complaint (days)	16 ± 4.0	12.4 ± 7.4	0.299	
Initial vision (LogMAR)	0.86 ± 0.84 (Snellen=20/160)	1.4 ± 1.0 (Snellen = 20/500)	0.117	
Anterior chamber reaction	1+ 7	1+ 3	0.154	
	2+ 2	2+ 7		
	3+ 4	3+ 4		
	4+ 0	4+ 1		
Keratic Percipitates	Yes 5	Yes 10	*P*=0.133	OR=0.577
	No 8	No 5		
Phakic status	Phakic 11	Phakic 12	*P*=0.572	OR=0.377
	Pseudophakic 2	Pseudophakic 2		
Lens status	Clear 7	Clear 11	*P*=0.131	OR=0.159
	Cataract 4	Cataract 1		
Degree of vitritis	1+ 5	1+ 0	*P*=0.007*	OR=3.825
	2+ 5	2+ 6		
	3+ 3	3+ 6		
	4+ 0	4+ 3		
Pattern of retinitis	Focal 3	Focal 0	*P*=0.010*	OR=1.04
	Multifocal 6	Multifocal 3		
	Diffuse 4	Diffuse 12		
Extent of retinitis (clock hours)	8 ± 3.8	11.5 ± 3.4	*P*=0.016*	
Axial Length (mm)	23.51 ±1.0	23.4 ± 0.6	*P*=0.794	
Viral etiology	HSV1 4	HSV1 3	*P*=0.511	
	VZV 7	VZV 8		
Intravitreal antiviral injection	Yes 7	Yes 13	*P*=0.055	OR=2.6
	No 6	No 2		
Prophylactic laser	Yes 5	Yes 7	*P*=0.662	OR=0.857
	No 8	No 8		
Previous ocular surgery	Yes 1	Yes 3	*P*=0.353	OR=1.5
	No 12	No 12		

**Table 5:** Analysis of possible risk factors for worse visual outcomes

Factor	*P* value
Age	0.583
Gender	0.035*
Immune status	0.287
Duration of symptoms	0.960
Initial vision	0.009*
PCR result	0.395
RRD	0.031*
Intravitreal antiviral treatment	0.069

## Discussion

This paper summarizes the clinical findings, management outcomes and complications of ARN in 28 eyes. To the best of our knowledge, the risk factors of RRD in ARN and the outcomes of RRD management in Saudi Arabia and the gulf region are not well delineated in the literature.

Regarding the viral etiology of ARN in this cohort, there was no difference in age between patients who had HSV (43.8 years) and VZV (41.8 years). As shown in the results, all patients who had HSV were found to have the HSV1 subtype which usually affects the older population, unlike the HSV2 subtype which tends to affect younger patients [2, 9–11]. Although there was a slight male predominance in our cohort (61.5%), the general consensus of no gender predilection in ARN stands still [9, 11]. The unilateral presentation (92.3%) distinguishes ARN from the progressive outer retinal necrosis (PORN) which mainly affects the immunocompromised [3, 11]. Although the most common cause of ARN was VZV (68.2%) which is known from the previous literature [12–14]. One patients had herpetic encephalitis which is a well-known risk factor for ARN [9]. Two of the 3 eyes were for one patient who developed sequential ARN and was treated with systemic and intravitreal antiviral and ended up with 20\25 vision in both eyes, while one eye was affected in an immunocompetent patient. Intravenous acyclovir remains the gold standard of treatment of ARN [6, 15], which was the treatment of choice. Timely initiation of the systemic antiviral therapy can help not only stop the progression of ARN and reduces the risk of complications, but also preventing further attacks in the fellow eyes [11, 16]. Prolonged antiviral therapy for ARN patients seems to reduce the risk of contralateral involvement further [17]. However, it is difficult to predict the time to discontinue the systemic antiviral treatment. Further consideration is also related to the onset of the systemic steroids to reduce the destructive effects of inflammation on the eye, while allowing the systemic antivirals to reach the therapeutic levels on the cellular level [18]. As is shown in the long term follow up of this cohort, two patients developed ARN in the contralateral eyes. One patients was not on systemic antiviral therapy, while the other was on a 1000 mg prophylactic dose of valacyclovir daily. Because our center is a tertiary care center, the duration between the onset of symptoms and hospital presentation was 14 days on average. Earlier presentation and initiation of systemic antiviral therapy might have resulted in better visual outcomes and less complication rates.

RRD developed in 57.1% of eyes. The incidence of RRD in ARN was variable in the literature (30–75%) because of the variable treatment modalities and the progressive nature of the disease which resulted in difference according to the timing of initiation of the systemic antiviral treatment [9, 13, 19–22]. Some studies have looked at possible risk factors of RRD in eyes which had ARN [9, 23, 24]. Butler et al. have found that the risk of RRD was significantly higher in eyes with retinitis involving 25% of the retina or more [9]. In addition, they have observed significantly lower prevalence of RRD among eyes which were treated with oral induction therapy and they explained this by indication bias as intravenous antiviral was administered only in aggressive presentations. On the other hand, Risseeuw et al. have found that there was no relationship between the extent of retinal involvement and the risk of RRD [23]. In addition, they have found marginally significant relationship between the use of intravitreal antiviral injections with higher risk of RRD development. Fitoussi et al. have found that the risk of RRD was significantly higher in eyes with more severe vitritis on presentation, while there was no relationship between the number of quadrants involved and the risk of RRD development [24]. The risk of developing RRD in our study was significantly higher in eyes which had higher grades of vitritis (*P* = 0.007, OR = 3.852), diffuse retinitis (*P* = 0.010, OR = 1.04) and the extent of retinitis (*P* = 0.016). There was marginally significant relationship between the administration of intravitreal antiviral injection and the risk of RRD (*P* = 0.055). The increased risk of RRD in eyes which had diffuse retinitis and more extensive retinal involvement (11.5 vs. 8 clock hours) indicates that the percentage of retinal involvement alone might not be adequate to assess the risk of RRD development, which also explains the inconsistent relationships between the extent of retinal involvement and the risk of RRD development in previous studies. A multifocal retinitis lesion which can be summed up to more than 3 clock hours (> 25% of retina) might not produce the cytotoxic and atrophic retinal changes which result from confluent or diffuse retinitis. These findings indicate the importance of documenting the retinitis pattern in addition to the extent of retinal involvement for more accurate assessment of RRD risk. The associations between severe vitritis and diffuse retinitis with the higher risk of RRD development show that the severity of the clinical picture of ARN plays an important role in the development of RRD. The observations of lower prevalence of RRD among ARN eyes which were treated with oral vs. intravenous antiviral and treated with intravitreal antiviral injections vs. eyes which were not treated with intravitreal antiviral injections further support that eyes with more severe clinical picture on presentation which warranted more aggressive treatment were at higher risks of RRD development. These results indicate that timely management of ARN might play an important role in reducing the risk of RRD by halting the progression of the clinical picture. There were no significant relationships between duration of symptoms, severity and signs of anterior chamber reaction, axial length, the viral etiology, prophylactic laser and prior intraocular surgeries with the development of RRD. When RRDs develop in eyes which had ARN, they are severe and challenging to repair. The incidence of total RRD in eyes with ARN is known to be high [19]. In this study, four eyes out of the 15 which developed RRD had inoperable total RRD, while the combination of scleral buckling (SB) with PPV was needed in 5 out of 11 eyes. In addition, silicone oil was used as a tamponading agent in the vast majority of operated eyes due to the complexity of RRD. The frequent use of silicone oil is known in eyes with RRD developed after viral retinitis [19, 25–27]. The role of prophylactic laser in ARN is suggested to be beneficial [28–30]. On the other hand, various studies showed that prophylactic laser has no benefit in these patients [9, 13, 23]. The results of this study show that RRD developed in 58% of eyes which had prophylactic laser and the role of prophylactic laser needs to be carefully considered.

The visual outcomes of ARN are variable and depend also on the duration between onset and initiation of therapy and the severity and progression of the disease [9, 11]. Our findings show that the male gender, lower initial vision and the development of RRD were significantly associated with worse visual outcomes. In our cohort, the male patients were more likely to develop total inoperable RRDs which have led to no light perception (NLP) vision. In addition, they were more likely to have other systemic diseases which complicate decisions for surgical interventions including herpetic encephalitis and pulmonary embolism. In eyes which received intravitreal antiviral treatment, there was marginally higher risk for worse visual outcomes (*P* = 0.069). This might be explained by the higher rates of RRD among these eyes due to the more severe clinical picture. Our findings indicate that the use of intravitreal antiviral treatment in eyes with aggressive ARN clinical pictures might not have significant role in preventing worse visual outcomes.

This study has potential limitations. Its primary design was that of a descriptive study, data were collected retrospectively and limited number of patients. When interpreting the results, the possibility of underreporting of cases, bias, or data loss must be considered. Given the rarity of ARN, the limitations are to be expected. Another limitation was the lack of viral load monitoring in this cohort. To the best of our knowledge, this is the first study to describe the clinical picture at presentation, method of diagnosis and to evaluate the course, visual outcome and the potential complications as well as treatment strategies of ARN in Saudi Arabia and the gulf region. The other strength of this study is the long duration of follow up for ARN and its complications.

## Conclusion

In conclusion, despite the advances in treatment of ARN, the visual outcomes are still variable, due to the progressive nature and serious complications of the disease. Early diagnosis and prompt initiation of antiviral treatment might be critical in the management of these cases. More severe posterior segment inflammation confers a higher risk of RRD. Management of RRD is challenging and visual outcomes are significantly lower than eyes which did not develop RRD.

## Supplementary Information

Supplementary Material 1: Supplemental figure 1. Kaplan Meie survival curve of the probability of developing RRD in eyes with acute retinal necrosisin relation to the grade of vitritis

Supplementary Material 2: Supplemental figure 2. Kaplan Meie survival curve of the probability of developing RRD in eyes with acute retinal necrosis (ARN) in relation to the severity of retinitis

## Data Availability

No datasets were generated or analysed during the current study.
