# Supplementary figures and images for: Clinical findings, outcomes following management and complications of acute retinal necrosis: the experience of a tertiary eye centre in Saudi Arabia
Source: J Ophthalmic Inflamm Infect. 2025 Jul 1;15:51. doi: 10.1186/s12348-025-00511-8 (PMC12214190; doi:10.1186/s12348-025-00511-8)

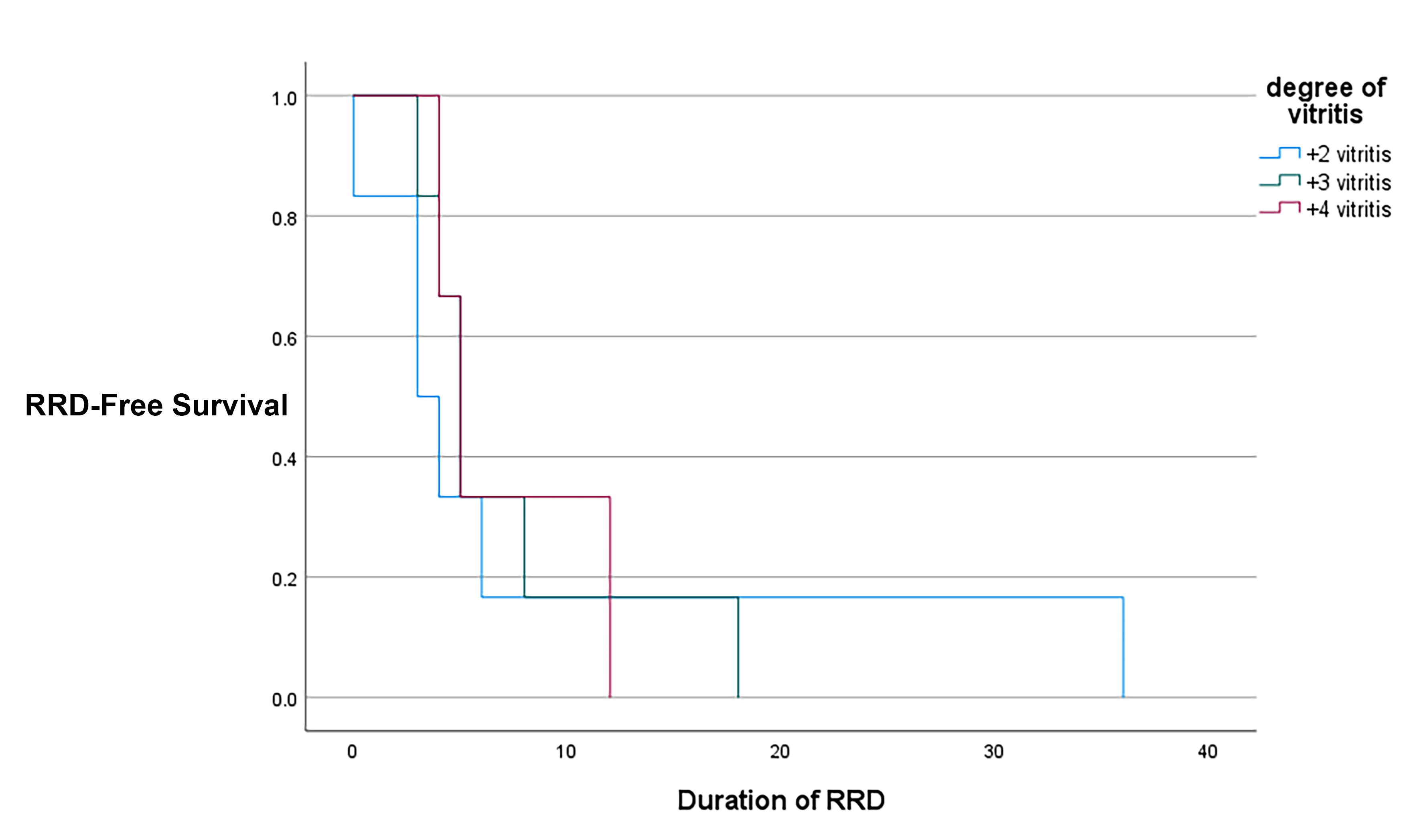

Supplement: Supplementary file 1 — Supplementary Material 1: Supplemental figure 1. Kaplan Meie survival curve of the probability of developing RRD in eyes with acute retinal necrosisin relation to the grade of vitritis [file 12348_2025_511_MOESM1_ESM.jpg]

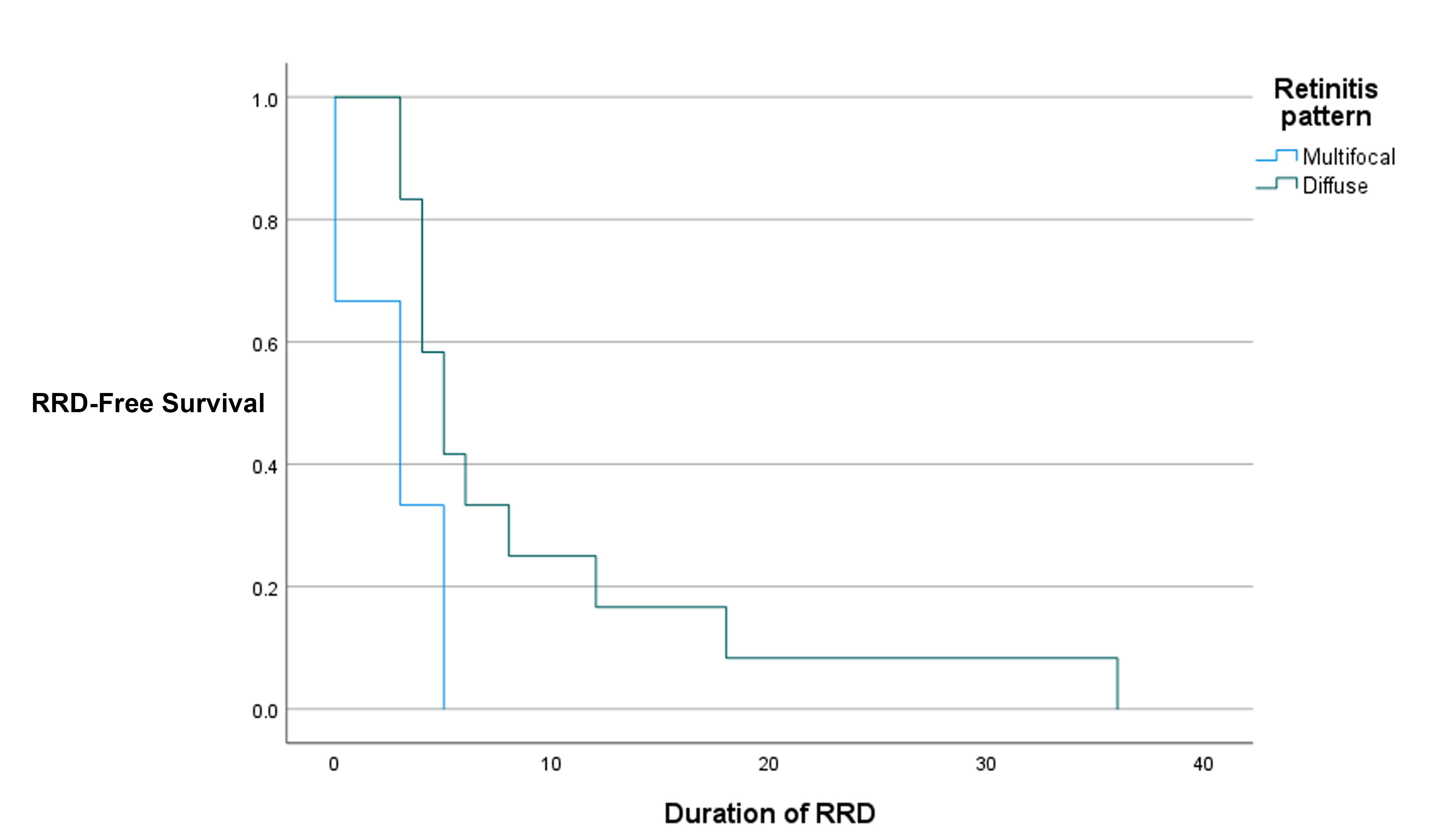

Supplement: Supplementary file 2 — Supplementary Material 2: Supplemental figure 2. Kaplan Meie survival curve of the probability of developing RRD in eyes with acute retinal necrosis (ARN) in relation to the severity of retinitis [file 12348_2025_511_MOESM2_ESM.jpg]
